# Replication Timing of Human Telomeres is Conserved during Immortalization and Influenced by Respective Subtelomeres

**DOI:** 10.1038/srep32510

**Published:** 2016-09-02

**Authors:** Laure Piqueret-Stephan, Michelle Ricoul, William M. Hempel, Laure Sabatier

**Affiliations:** 1 PROCyTOX Commissariat à l′Energie Atomique et aux Energies Alternatives (CEA), Fontenay-aux-Roses and Université Paris-Saclay, France

## Abstract

Telomeres are specific structures that protect chromosome ends and act as a biological clock, preventing normal cells from replicating indefinitely. Mammalian telomeres are replicated throughout S-phase in a predetermined order. However, the mechanism of this regulation is still unknown. We wished to investigate this phenomenon under physiological conditions in a changing environment, such as the immortalization process to better understand the mechanism for its control. We thus examined the timing of human telomere replication in normal and SV40 immortalized cells, which are cytogenetically very similar to cancer cells. We found that the timing of telomere replication was globally conserved under different conditions during the immortalization process. The timing of telomere replication was conserved despite changes in telomere length due to endogenous telomerase reactivation, in duplicated homologous chromosomes, and in rearranged chromosomes. Importantly, translocated telomeres, possessing their initial subtelomere, retained the replication timing of their homolog, independently of the proportion of the translocated arm, even when the remaining flanking DNA is restricted to its subtelomere, the closest chromosome-specific sequences (inferior to 500 kb). Our observations support the notion that subtelomere regions strongly influence the replication timing of the associated telomere.

Telomeres are specific nucleoprotein structures comprised of TTAGGG nucleotide repeats, located at the end of chromosomes^1^. Telomeric DNA is primarily double stranded, with the exception of the 3′ end, as the strand is longer, and forms a loop called the T-loop^2^,^3^,^4^. This particular structure allows telomeres to protect chromosome ends from degradation and fusion. Indeed, telomere structures protect chromosome ends from being recognized as double strand breaks (DSB), and recruitment of the DSB repair machinery. Two distinct domains composed of more or less degenerate telomeric pattern repeats called subtelomere regions are located upstream of telomeres. The two subtelomere domains are defined by their position with respect to the centromere: proximal and distal^5^. Subtelomeres are transitional regions between the telomeric repeats of the chromosome ends and the rest of the chromosomes. They are composed of 10–300 Kb of variable sequence and are unusually dynamic. These blocks of sequence are relatively heterogeneous with respect to size (some are small whereas others can reach more than 50 kb) and repeatability, but share from 90% to more than 99.5% sequence similarity^6^,^7^. Contrary to telomeres, which are identical for all chromosome ends; distal subtelomere regions are specific for each arm of each chromosome.

In somatic cells, telomere length decreases with each cellular division, due to the inability to completely replicate the end of the linear chromosome by DNA polymerase^8^. After a fixed number of cellular divisions^9^, they arrive to a critical size that leads to metabolic arrest of the cell called replicative senescence^10^. Telomeres act, thus, as a biologic clock. In stem, germ, or cancer cells, this function is abrogated and telomere length is maintained by a specialized protein called telomerase^11^,^12^. The majority of cancer cells express telomerase, but telomere maintenance for 7–10% is performed by an alternative mechanism called Alternative Lengthening of the Telomere (ALT)^13^,^14^.

Telomeric structures and functions are well conserved during evolution, but their replication timing is subject to species-specific differences. Indeed, mammalian telomeres replicate during specific time windows throughout S-phase (Synthesis phase)^15^,^16^,^17^, whereas yeast telomeres replicate only during late S-phase^18^. In addition, yeast telomeres replicate earlier than normal when they are short^19^,^20^, whereas no correlation between telomere length and their replication timing has been observed in humans^17^. In mammalian cells, telomere replication of opposite chromosome arms (i.e. p- and q-arms) is asynchronous, while that of homologous chromosomes occurs in a synchronous fashion^16^. Furthermore, replication timing of individual telomeres appears to be conserved in humans and is associated with nuclear localization. Indeed, telomeres found at more internal positions are replicated early, whereas those that are more peripheral are replicated late^17^. An artificial system has provided further evidence of this phenomenon by the insertion of a D4Z4 repeated sequence, alone, or associated with a β-satellite sequence, into the subtelomeric region. This leads to a delay in the replication timing of the associated telomere and shifts it to a more peripheral radial position in the nucleus^17^. Such mechanisms have also been observed in Ku-deficient yeast, where telomeres relocate from the nuclear periphery leading to earlier replication^21^. In chimpanzees, the presence of a heterochromatin cap at the subtelomere region delays the replication timing of the associated telomere^22^. Despite these and other studies, the factor(s) that governs the timing of telomere replication remains unknown.

During cellular immortalization, chromosomes undergo a large number of modifications^23^,^24^. Indeed, they are subject to duplication and rearrangement until the cells obtain a proliferative advantage. In addition, the lengths of the chromosome ends are modified due to endogenous telomerase reactivation^12^. Thus, cellular immortalization is a useful model to investigate telomere replication in a constantly changing environment. We used SV40 immortalized cells because this is a good model for cancer cells as they share the same types of chromosomal rearrangements, and chromosomal and chromosomal arm imbalances. Using this model, we studied telomere replication timing at different population doublings during cellular immortalization in different types of chromosomes: normal, duplicated, or following translocation in the presence of the endogenous telomerase activity. Our approach was to analyze the replication status under physiological conditions in the context of chromosome rearrangement. We thus used a derivative of the CO-FISH approach^25^: the ReD-FISH (Replicative Detargeting-FISH) technique described by Zou *et al.*^16^ to analyze replication timing of individual telomeres under these different conditions. Our observations underscore the fact that subtelomeric regions strongly influence telomere replication timing, regardless of the state of chromosome, and despite the high level of instability observed during cellular immortalization by SV40 transformation.

## Results

### Model studies

**Figure 1:**
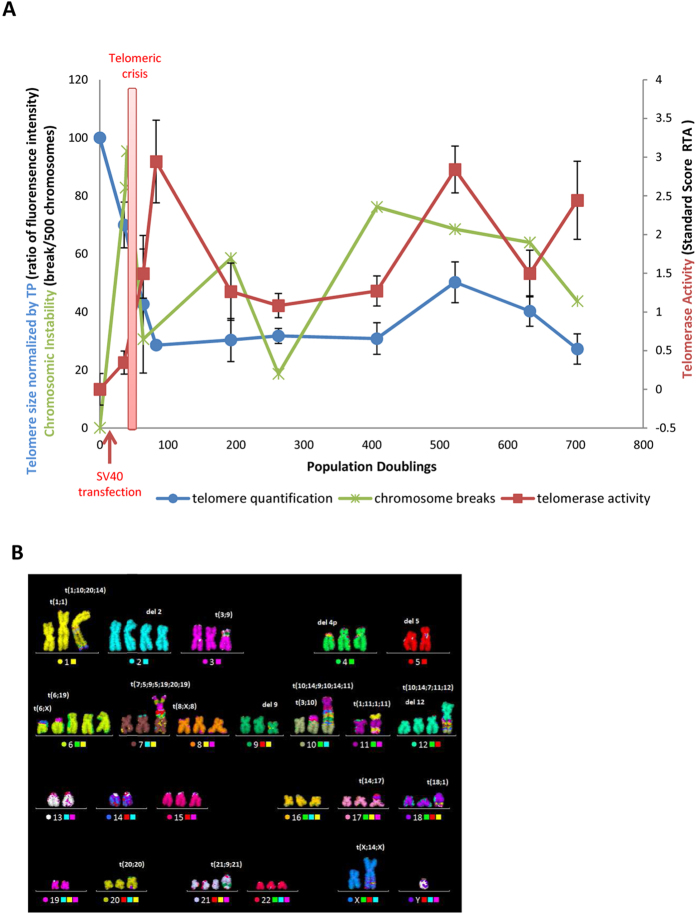
Immortalization of TP fibroblasts following SV40 transfection. (**A**) Evolution of mean telomere length (blue circles), the frequency of breaks per 500 chromosomes (green crosses) and telomerase activity (red squares) during cellular immortalization. (**B**) Example of chromosomal rearrangements in the TP 15.5 PD523 karyotype.

Primary human fibroblasts (TP) were transfected with SV40LT. A subclone, which we named TP15.5, emerged after crisis at population doubling (PD) 36 from this transfection. We analyzed several parameters of subclone TP15.5 at different PD throughout the process of TP cell transformation, (PDs 36, 64, 83, 193, 263, 408, 523, 633, and 703). We compared telomere length, telomerase activity and chromosomal instability at the different PDs to those of the primary fibroblasts TP (PD “0”), pre-immortalization, chosen as the starting point of the study (Fig. 1A). These analyzes showed that SV40 transfection induced telomeric crisis, noted by a dramatic decrease in telomere length during the early PD until reaching a critical size. This was followed by length stabilization at the end of the crisis at PD83, consistent with endogenous telomerase reactivation. We also observed that endogenous telomerase reactivation underwent fluctuations during cellular immortalization. SV40 transfection also induced chromosomal instability with a peak of chromosomal rearrangement during crisis, where most of the cells die associated with telomeric crisis^23^, until they obtain a proliferative advantage. Karyotype analysis of these cells during the last PDs revealed that chromosomal instability led to, not only simple, but also complex chromosomal rearrangements (Fig. 1B).

We used TP15.5 cells to compare the timing of telomere replication during cellular immortalization to that of the primary cells. We selected four population doublings corresponding to the control cell TP (PD “0”), early telomerase reactivation (PD 64), telomerase activity stabilization (PD 263) and, finally, the fluctuation of telomerase activity (PD 523). We thus chose to focus on the characterization of chromosome rearrangements during the immortalization process, focusing on their accumulation, throughout the population doublings. We also analyzed four important stages of the same sub-clone throughout the immortalization process and its primary cells instead of studying different cell lines.

### Cell immortalization leads to a modification of S-sub-phase lengths

**Figure 2:**
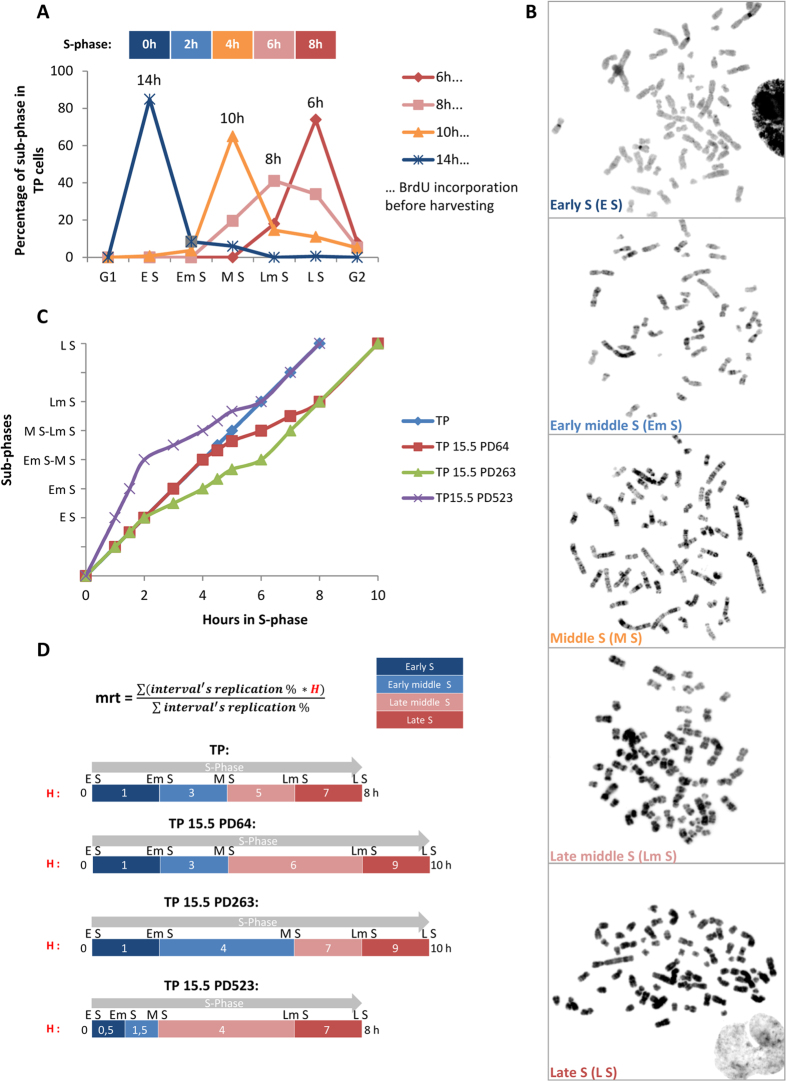
Variable length of S-phase and of its sub-phases during cellular immortalization. (**A**) Example in TP cells of S-phase length determination with the aid of the observed distribution of the sub-phases at each BrdU incorporation time. The five S-sub-phases are in temporal order: Early S (E S), Early middle S (Em S), Middle S (M S), Late middle S (Lm S) and Late S (L S). (**B**) Reverse DAPI staining of metaphase spreads following ReD-FISH for the five S-sub-phases. (**C**) Summary of the length of each sub-phase for the control cells (TP) and for each population doubling studied (TP 15.5 PD64, PD263 and PD523). (**D**) To classify telomeres as a function of their replication, the mean replication timing (mrt) was calculated. It is a weighted average based on the percentage of single telomere replication for each of four periods (i.e. the intervals of the five sub-phases) and the length of each sub-phase. For the length of each sub-phase, a time indicator H corresponding to the mean hour of the sub-phase interval was used. Thus, this calculation is specific to individual telomeres and the population doubling.

To study the timing of telomere replication, it was first necessary to determine whether the length of the entire S-phase and of its sub-phases are conserved; or vary as a function of the PD. We therefore examined this parameter for each PD studied, with the aid of BrdU incorporation (Fig. 2A–C), to allow the comparison of the replication timing between different PDs. We determined that the length of the S-phase of the primary TP cells was eight hours. This analysis showed that the length of the entire S- phase increased by two hours following SV40 transfection (PDs 64 and 263), before returning to the original duration of eight hours (PD 523). Interestingly, cellular immortalization also induced a variation in S-sub-phase length even when the length of the entire S-phase was the same as the control cell (e.g. PD 523). Indeed, the length of each of the S-sub-phases of the control cell TP was the same (two hours per sub-phases) (Fig. 2A), whereas some sub-phases were longer (e.g. four hours for PD 64 from Middle S to Late middle S) or shorter (e.g. one hour for PD 523 from Early S to Early middle S) (Fig. 2C).

These differences necessitated the use of a specific calculation for determining the precise telomere Mean Replication Timing (mrt) for each population doubling (Fig. 2D) as described in the Material and Methods.

### The replication timing of human telomeres is conserved among individuals

**Figure 3:**
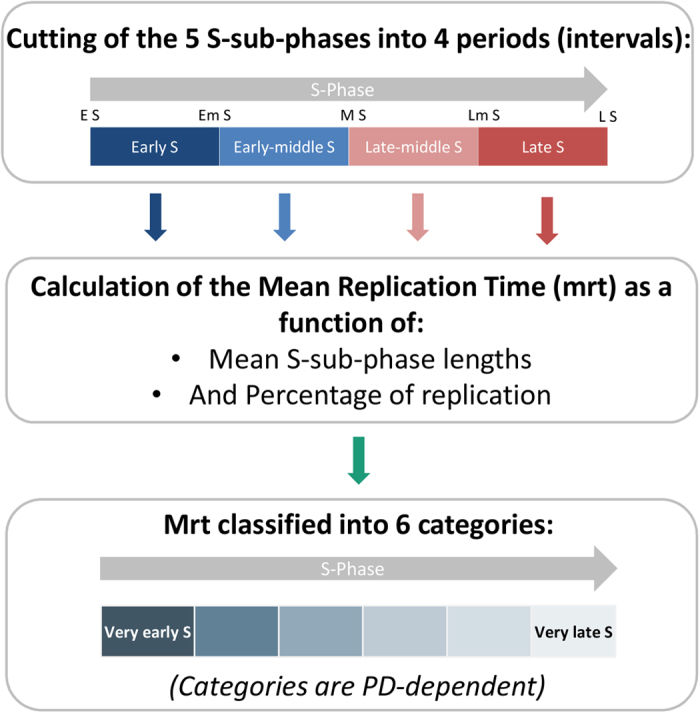
Flow chart of the telomere replication timing analysis. First, the 5 S-sub-phases are divided into 4 periods, corresponding to the 5 S-sub-phase intervals: (Early S (E S), Early middle S (Em S), Middle S (M S), Late middle S (Lm S) and Late S (L S)). Second, the mean replication timing is calculated as a function of the length of each period (PD-dependent) and the percentage of replication in each period: more details are provided in Fig. 2D for each telomere for each PD studied. Third, each telomere is classified according to its mrt into 6 categories (from a replication timing of very early S to very late S, hour-dependent). These categories are PD-dependent as they depend on S-Sub-phase length. This system allows the comparison of each telomere according to its replication status between PDs and chromosomes.

**Figure 4:**
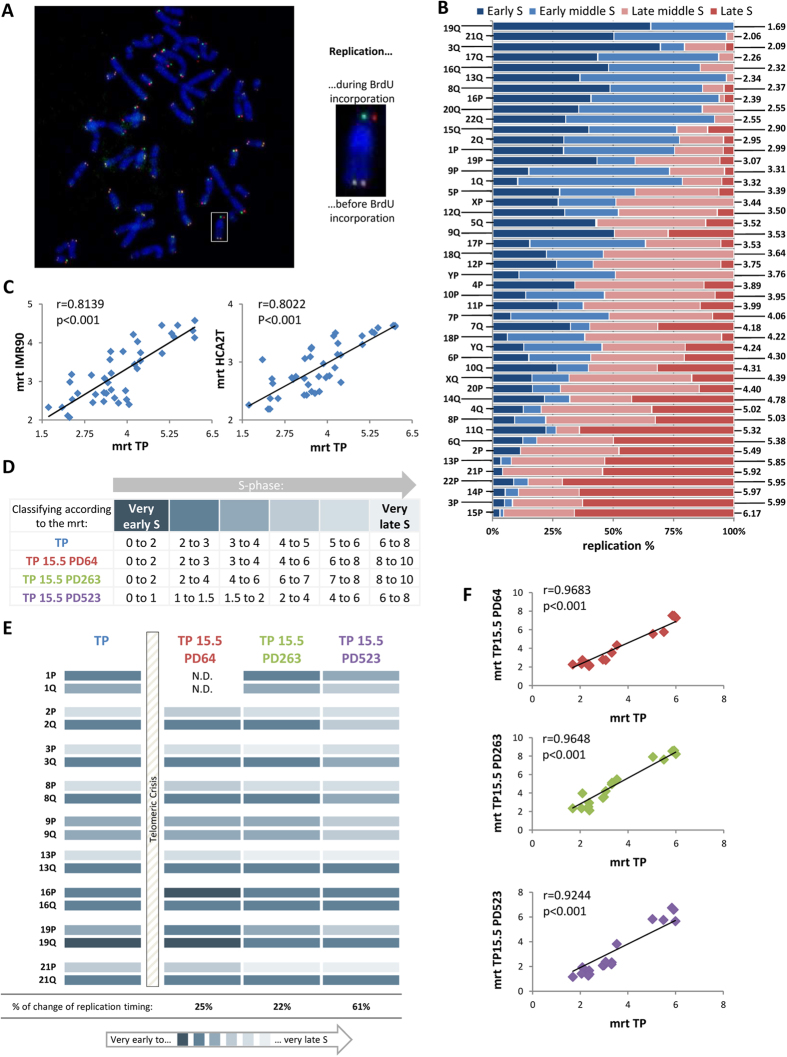
Replication timing profile of human telomeres is conserved between individuals and during immortalization. (**A**) ReD-FISH on TP metaphases. This hybridization uses two different telomere probes (CCCTAA-Cy3, TTAGGG-FITC). The chromosome in the white box was enlarged as an example. It has a detargeted telomere on the p-arm, (one probe per chromatid) and a non-detargeted telomere on the q-arm, (the two probes per chromatid), signifying that the telomere of the p-arm was replicated after BrdU addition unlike the telomere of the q-arm which was replicated before. (**B**) Replication timing of every single telomeres in TP cells. Horizontal bars represent the percentage of replication during each of four periods (Early-S, Early-middle-S, Late-middle-S and Late-S) and are normalized to 100%. Single telomeres are listed in ascending order of mrt (right). (**C**) Significant correlation between mrt of single telomeres in TP cells and IMR90 cells (left) and HCA2T cells (right), indicating the global conservation of replication timing of human telomeres. Mrt values of IMR90 and HCA2T correspond to those previously published^14^ (Spearman’s rank correlation coefficient). (**D**) Summary of telomere classification as a function of their replication. Telomeres were classified into six categories, represented by the color code, according to their mrt (in hour). As the length of the different sub-phases was not constant during immortalization, the categories were not equal between the different population doublings. (**E**) Pattern of single telomere replication during immortalization (TP as a control and PD64, 263 and 523 of TP15.5). The single telomeres studied for this study are those of both arms of chromosomes 1, 2, 3, 8, 9, 13, 16, 19 and 21. Telomeres are classified according their mrt as described in D. The percentage change of replication timing in each PD compared to the control TP is indicated below. (**F**) Significant correlation between the mrt of single telomeres in TP and of those of the different PD studied of TP 15.5 (PD64, PD263, and PD523 in red, green and purple respectively) for the 18 telomeres studied indicating that the relative order of telomere replication timing is not modified during immortalization (Spearman’s rank correlation coefficient).

The global flow chart of the telomere replication timing analysis is shown in Fig. 3. For that study, we used the ReD-FISH technique^16^, a variation of the CO-FISH technique^25^ (Fig. 4A). We first determined the replication of all telomeres of the control cell TP, with the aid of Multi-FISH to distinguish the chromosomes. A mrt was determined for each individual telomere as described in the Material and Methods (Fig. 4B, SD1). The homologous telomeres data were pooled for the mrt calculation as homologous chromosomes are replicated synchronously in mammalian cells^16^,^17^.

A previous study showed that the pattern of individual telomere replication was conserved between cells from different individuals^17^. We compared our telomere replication profile of primary TP cells with those of the cells used in the other study (IMR90 and HCA2T) to further corroborate this observation. The analyses of the two studies were carried out somewhat differently in that we took into consideration the length of each S-sub-phase whereas they did not. For primary TP cells, the length of each S-sub-phase was the same (two hours each); thus, the overall difference in these two analyses is minimal. We found a significant correlation (Spearman’s rank correlation) for the mrt between telomeres of our primary TP control cells and the telomeres of their cell lines (Fig. 4C). We thus reinforce the finding that the replication timing of human telomeres is conserved amongst individuals.

### Endogenous telomerase affects individual telomere replication, but not general telomere replication

To study whether endogenous telomerase reactivation *in vitro* could affect telomere replication during immortalization, we determined the telomere mrt at three PDs corresponding to three different states and levels of the protein activity and compared these to those of the control TP cell. To compare telomere replication from one PD to another, we had to, at first, classify them into six categories according to the length of the S-sub-phases for each PD and to their mrt for nine chromosomes (Figs 3 and 4D). The replication of all analyzed telomeres is summarized in Fig. 4E. These results show a small and similar change for the replication timing at PDs 64 and 263 (25% i.e. 4/16 and 22% i.e. 4/18 respectively), whereas a bigger change was observed at PD 523 (61% i.e. 11/18). Interestingly, cells of PDs 64 and 263 shared a similar and moderate level of telomerase activity, whereas cells of PD 523 showed a high level of telomerase activity and heterogeneity of telomere length (unpublished data).

Overall, the observed changes at all PDs were relatively minor. Indeed, none were drastic: no early replicating telomeres became late and vice-versa. In addition, the changes in replication timing were exclusively in the same direction. Immediately following telomeric crisis, at PD 64, telomere replication changes occurred exclusively earlier during S-Phase; whereas later, after telomeric crisis, at PDs 263 and 523, telomere replication changes occurred exclusively later during S-Phase.

We compared all analyzed replicated telomeres for each PD of TP15.5 with those of the control TP cells (Fig. 4F) to determine whether the observed changes were indicative of a modification of the global replication profile. We observed a significant correlation between the mrt of TP control cells and the other three PDs of TP15.5 (Spearman’s rank correlation), suggesting that the changes in replication observed for individual telomeres are not sufficient to change the global replication profile. This is most certainly due to the fact that the changes of replication observed for each PD were always in the same direction.

### Telomere replication at duplicated homologous chromosomes is synchronous

**Figure 5:**
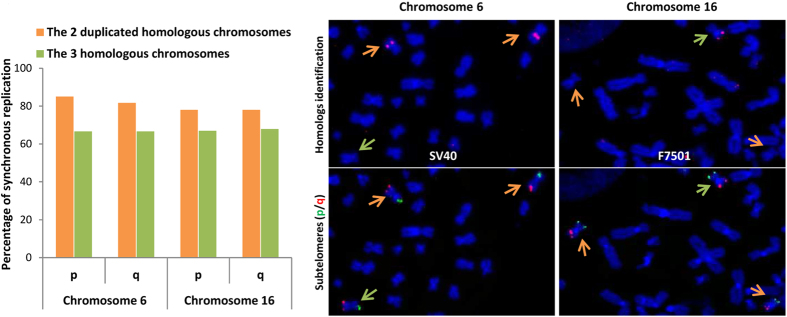
Strong conservation of the replication timing between the duplicated homologous chromosomes. Telomeres of both arms (p/q) of the two duplicated homologous chromosomes (orange) are more synchronous than those of the three homologous chromosomes (green) for both chromosomes 6 and 16 during the entire S-phase in TP15.5 PD523. The two duplicated homologs of chromosome 6 can be distinguished by SV40 hybridization (the two duplicated chromosomes 6 out of the three are labeled by the SV40 plasmid, cf. supplementary data 2). The two duplicated homologous of chromosomes 16 can be distinguished by the F 7501 subtelomeric probe (one of the three chromosomes 16 is labeled by this probe, and the two duplicated chromosomes are not). Telomere replication for duplicated homologous chromosomes and for the three homologous chromosomes is not random (chi-squared test). The test of the two duplicated chromosomes was much stronger than for the three homologous chromosomes (p-value ≈ 10-10 Vs. 10-4 on average), confirming that telomere replication for the duplicated homologous chromosomes is more synchronous than that for the three homologous chromosomes.

Previous studies have shown that telomeres of homologous chromosomes are replicated synchronously^16^,^17^. In cancer cells, some homologous chromosomes can be found in more than two copies in the case of polyploidy or after a duplication event. To determine whether telomeres of duplicated homologous chromosomes are also synchronously replicated, we examined telomere replication of chromosomes 6 and 16 of our cell lines (Fig. 5A). It is noteworthy that SV40 transformed cells are a good model for cancer cells as they share the same types of chromosomal rearrangements, and chromosomal and chromosomal arm imbalances. Indeed, these chromosomes, 6 and 16, are found in three copies at the late population doublings analyzed and each of the homologous chromosomes can be distinguished due to DNA polymorphisms (Figure SD2). This analysis showed that telomeres of duplicated homologous chromosomes are also synchronously replicated (≈80%), and to a much greater degree than the three homologous chromosomes (≈60%). The chi-squared test indicates that replication synchronicity is more highly significant for the duplicated homologous chromosomes (≈10^−10^) than for all three homologous chromosomes (≈10^−4^); and that neither result is random.

### Replication timing of translocated telomeres is conserved

**Figure 6:**
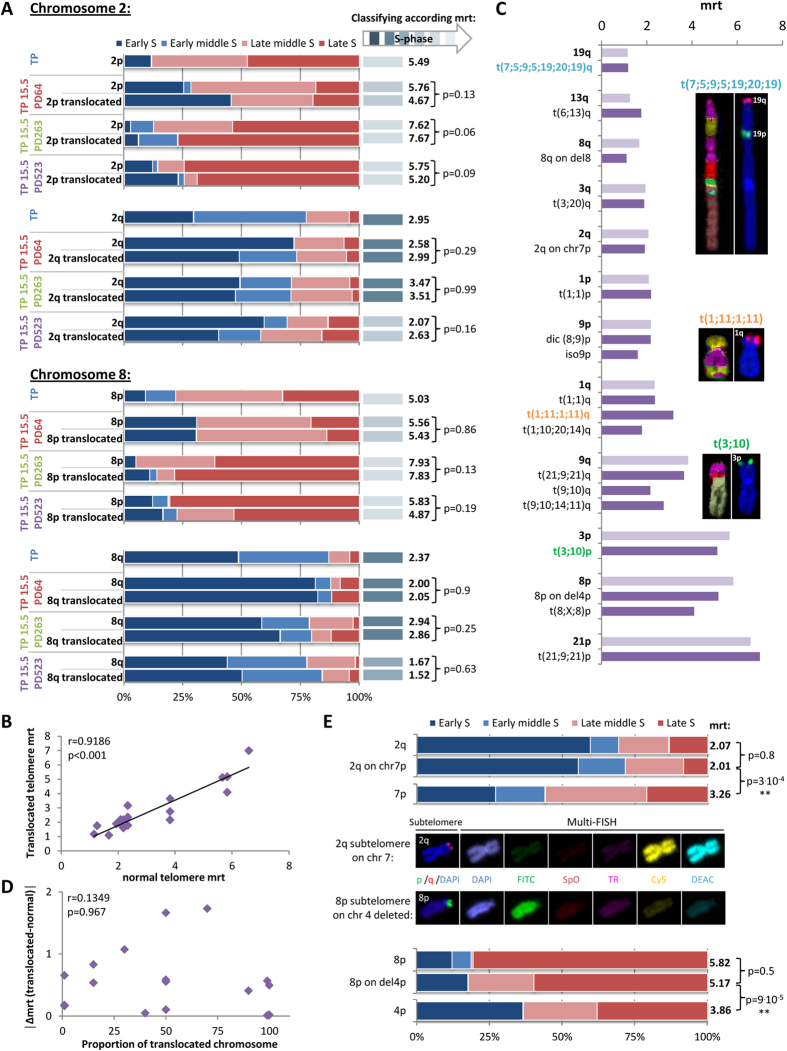
Translocated telomeres possessing their initial subtelomere retain their replication timing. (**A**) Replication timing of telomeres of both arms of chromosomes 2 and 8 in all PD studied (i.e. TP as a control cell and PD64, PD263 and PD523 of TP15.5) for normal and rearranged chromosomes. Telomeres are classified according to their mrt (right). Translocated telomeres have the same replication timing as telomeres from normal chromosomes even if they temporarily change their replication timing in some PD (at PD64 for telomeres 2p and 8p, and at PD523 for telomeres 2q and 8q), (Fisher exact test). (**B**) Significant correlation between mrt of normal and translocated telomeres in TP15.5 PD523 (Spearman’s rank correlation coefficient), indicating that telomere replication timing is not modified by the translocation of telomeres onto another chromosome. (**C**) Examples of the comparison of mrt of normal and translocated telomeres in PD523 of TP15.5. Telomeres are listed in ascending order by normal telomere mrt. t(7;5;9;5;19;20;19) and t(1;11;1;11) as a complex chromosomal rearrangement and t(3;10) as a simple chromosomal rearrangement are represented. Translocated telomeres (dark purple) appear to have generally the same replication timing as normal ones (light purple). (**D**) No correlation between the length of translocated arms and the difference of mrt between normal and translocated telomeres, indicating that the length of the translocated arm does not affect the mrt of the translocated telomeres (Spearman’s rank correlation coefficient). (**E**) Comparisons of replication timing between a normal telomere and a telomere translocated only with its subtelomere in TP15.5 PD523: (i) telomere 2q, translocated telomere 2q on chromosome 7, and normal telomere 7p (upper panels), (ii) telomere 8p, translocated telomere 8p on chromosome 4 deleted and normal telomere 4p (lower panels). Images of these chromosomal rearrangements were captured (middle panels) by hybridization with subtelomere specific probes (p-arm in FITC and q-arm in TexasRed) and Multi-FISH (combination of Cy5 and DEAC label corresponds to chromosome 7, and a single label of FITC corresponds to chromosome 4). Telomeres translocated along with their subtelomeres onto another chromosome follow the replication timing of normal telomeres (Fisher exact test).

In cancer cells, chromosomal rearrangements are frequent. We examined the replication timing of translocated telomeres by ReD-FISH and subtelomeric hybridization to determine the effect of translocation of the telomere to another chromosome on replication timing. Replication of all chromosomal rearrangements comprising telomeres of chromosomes 2 and 8 on both arms (p and q) for each of the studied population doublings are represented in Fig. 6A. For this study, approximately 20 metaphases for translocated telomere 2p, 30 for translocated 2q, 55 for translocated 8p, and 30 for translocated 8q were analyzed per period, and all translocations (sporadic or clonal) merged. These results led to two major conclusions. First, translocated telomeres retain the replication timing of telomeres found on the normal chromosomes at the PD studied (Fisher exact test). Second, when telomeres located on normal chromosomes temporarily change their replication timing at a certain PD, translocated telomeres also change the replication timing in the same way (Fisher exact test), as observed for telomeres 2p and 8p at PD 64 and for telomeres 2q and 8q at PD 523.

To verify that these observations are generalizable to all chromosomal rearrangements, we analyzed the replication timing of telomeres found on 18 clonal chromosomal rearrangements at PD 523 comprising short, medium, and long translocated chromosomes. We carried out multi-FISH staining following ReD-FISH and subtelomeric hybridization to distinguish the different clonal chromosomal rearrangements. We observed a significant correlation between the mrt of telomeres localized on normal chromosomes and those on rearranged chromosomes (Spearman’s rank correlation) (Fig. 6B). The mrt of telomeres localized to the 18 clonal chromosomal rearrangements analysed for the nine chromosomes studied are shown in Fig. 6C and compared to the mrt of telomeres of normal chromosomes. These data clearly demonstrate that telomeres associated with simple and complex chromosomal rearrangements share a similar mrt to telomeres of normal chromosomes.

### Subtelomere regions play an important role in the replication timing of human telomeres

We examined the correlation between the mrt of telomeres of rearranged and normal chromosomes at PD 523 and the proportion of the translocated chromosome present in the 18 clonal rearranged chromosome (Fig. 6D) to determine whether the length of the translocated chromosomal arm is important for determining replication timing. We observed no correlation between these two parameters (Spearman’s rank correlation). Thus, the length of the chromosomal arm carrying the translocated telomeres does not influence their replication timing. We further analyzed telomere mrt for the two cases with the shortest translocation, those involving the translocation of only the associated subtelomere. Thus, the replication of telomere 2q on the p-arm of chromosome 7 and telomere 8p on the p-arm of chromosome 4 deleted (Fig. 6E) were studied. Importantly, this analysis revealed similar replication throughout S-phase with no significant difference of the mrt between normal and translocated telomeres. In addition, the replication profile was different between the translocated telomere (2q and 8p) and the original telomere (7p and 4p respectively). Of note, the replication timing observed at the end of these chromosomes corresponded to that of the translocated and not the original telomere. Furthermore, there is no effect of centromere position, chromosomal morphology, or the length of the translocated arm. These results suggest that subtelomeric regions alone govern the replication timing of the associated-telomere.

## Discussion

Cancer cells present a large number of chromosomal rearrangements and chromosomal and chromosome arm imbalances. In this study we have analyzed the replication timing of human telomeres under physiological conditions using a cellular immortalization model which closely mimics the chromosomal instability and telomere changes observed in cancer cells to decipher the role of chromosomal regions in the regulation of telomere replication timing. Indeed, we observed a number of specific phenomena during SV40LT-induced immortalization in our model system. We observed a decrease of telomere length up to a critical point corresponding to the bypass of senescence and telomeric crisis. Chromosomal instability, induced by short telomeres, was thus induced during this crisis until the cells obtained a proliferative advantage. The surviving subclone is that which reactivates its endogenous telomerase activity and stabilizes or increases its telomere length. As these events directly involved the telomeres, we explored whether the timing of human telomere replication changed during this process or whether it was conserved. Our experiments show that telomerase activity correlated with slight variations on telomere replication but without significantly affecting the global replication profile; and that individual telomere replication timing was guided by its associated subtelomere. These observations support the notion that telomere replication is programmed and remains unchanged, independent of the state of the chromosome: duplication, rearrangement, translocation onto another chromosome. It may seem tempting to test the effect of turning on/off telomerase or to delete the subtelomeric regions. However, we did not want to induce nuclear reorganization, or a modification of telomere replication that would be purely artificial.

The global replication profile of the telomeres under study remained unchanged throughout the immortalization process, whereas some minor changes occurred at the individual level. The observed changes were always in the same direction for each population doubling (i.e. always earlier just after crisis, and always later after crisis), and did not appear to be random. Indeed, the rate of the modification at each PD was concordant with the level of telomerase activity: the greatest change of replication timing occured at the PD in which the cells had a high level of telomerase activity (i.e. PD 523). Thus, telomerase may interfere with the replication timing of telomeres resulting in the delay or advance of their replication. However, as the modifications were relatively minor, the telomere replication profile was globally conserved during cellular immortalization relative to the control cell line. Indeed, despite the consideration that the changes in S-phase and S-sub-phase length for each population doubling were integrated into the calculation of the mean replication timing used to classify telomere replication, such slight modifications were still observed.

Telomerase reactivation during the immortalization process implies a modification of individual telomere length. Indeed, the maintenance of global telomere length is performed by a combination of the elongation of short telomeres by telomerase and natural shortening of longer telomeres^26^,^27^,^28^,^29^,^30^,^31^,^32^. A comparison of individual telomere lengths for the different population doublings studied and their mrt showed that there was no correlation between those two parameters (Figure SD3). Indeed, the telomere length of homologous chromosomes was different in the cells under study unpublished data^33^, whereas their replication was synchronous^16^. Thus, telomere length had no impact on their replication timing as previously observed^17^. Individual telomere length was modified during the immortalization process contrary to global replication timing, which was not. This observation is concordant with the notion of the conservation of telomere replication timing between individuals, as single telomere length varies from one individual to another^34^,^35^. However, we found that in the early stage of the S-phase, the first telomere replicated amongst homologs were the longer ones for telomeres on p-arms (in 74% of cases) whereas there was no size preference for telomeres on q-arms (around 50% each) (data not shown) in TP primary cells. Our study shows that the replication order for homologous telomeres on q-arms is random whereas the replication order of telomeres on the homologous p arm is length dependent. The replication of telomeres on q-arms and p-arms are predominantly early and late in S-phase, respectively. We hypothesized that when a telomere is replicated before its mean replication timing, the longer telomere is replicated first due to the higher probability of the replication machinery encountering long telomeres.

As previously described^16^,^17^ and observed in our study, telomeres of chromosome homologs exhibit synchronous replication in mammals. Moreover, we show that this synchrony is even stronger in the case of chromosome duplication in humans, certainly due to the fact that they were subjected to evolutionary drift for less time than non-duplicated chromosomes. Strikingly, homologous chromosomes^36^ and duplicated homologous chromosomes localized far from each other, with the duplicated homolog finally coming to rest near the non-parental homolog (data unpublished) as it “tries” to put maximum distance between itself and its copy suggesting that chromosomal territories do not appear to influence telomere replication. In another study, it was concluded that there was a correlation between the mean replication timing of single telomeres and their mean volume ratio (i.e. their radial position)^17^. These findings are not contradictory. Indeed, both of these observations indicate that human telomeres do not need to be in proximal chromosomal territories to exhibit similar replication timing, but they need to be at the same radial position in the nucleus.

Finally, we show that translocated telomeres retain, in general, the replication timing of the normal telomere. Moreover, the proportion of the translocated chromosomal arm had no impact on the conservation of the replication timing of the telomere. Indeed, translocation of a telomere and its associated subtelomere is sufficient to maintain its replication timing even if no initial chromosome remains and even if they are present in a complex chromosomal rearrangement. Interestingly, a previous study showed that insertion of a macrosatellite repeat D4Z4 into the subtelomeric region delays replication of the associated-telomere^17^. Both results suggest that the subtelomeric region is important for controlling telomere replication timing, and, thus, a regulatory factor may be located within this specific region. This hypothesis is supported by a recent study that showed that the initiation of telomere replication occurs predominantly within the subtelomere or a region upstream of the subtelomere^37^. Our study confirms the role of subtelomere sequences in regulating telomere replication timing and furthermore, strongly supports the conclusion that neither centromeres nor other chromosomal sequences play a role in this process, and finally, that subtelomere sequences may be sufficient to determine telomere replication timing. Interestingly, even if translocated telomeres retained the timing of normal telomere replication in general, some small modifications were observed. Those modifications could be explained by the fact that the radial position in the nucleus influences replication timing. Indeed, the translocated telomeres may be shifted in the mean volume ratio, hence their radial position, due to chromosomal constraints, because of the complex rearrangements, resulting thus in a slight modification of their replication timing. As subtelomeric regions are separated from the associated-telomere by a degenerate telomeric region of 50 to 300 kb, this degenerate region could undergo mutation or cryptic translocations that could also explain the slight modifications observed on the timing of telomere replication. Indeed, modifications of telomere replication timing were very slight as observed on Fig. 4E, and these changes may have occurred due to alterations in the regulatory region between the subtelomere and its associated-telomere. The small changes that were observed may also simply be due to the limit of the technique.

In conclusion, we show that, under physiological conditions, human telomere replication timing is globally conserved under different situations: between individuals, during immortalization with endogenous telomerase reactivation, between duplicated homologs and, finally, when the telomere is translocated with its associated subtelomere onto another chromosome. This is the first study to examine the timing of telomere replication, under physiological conditions, in a changing environment such as during the immortalization process, thus taking into account native telomerase reactivation and chromosomal aberrations such as duplicated or rearranged chromosomes. In agreement with previous studies, these results, obtained on one subclone but for four very different states, due to cell derivation after SV40 transfection, show that human telomere replication timing is programmed and is strongly associated with their subtelomere. The factor that regulates telomere replication is still unknown but it appears to be associated with the subtelomere region. Recent studies showed that the protein Rif1 (Rap1-interacting-factor-1) regulates the replication timing domains of the human genome^38^,^39^; and of the yeast telomeres^40^,^41^,^42^. Furthermore, in the mammalian genome, this protein binds to the late-replicating domains and is associated with a structure implicated in the organization the nuclear architecture: Lamin B1, suggesting that Rif1 could be an organizer of the nuclear architecture related to the timing of the replication of chromatin organization in the nuclear volume. It was also shown that Rif1 coordinates the inter-domain interactions prior to S-phase^43^. Nevertheless, no study has yet correlated the activity of this protein with the regulation of telomere replication timing in humans. It would be interesting to confirm whether this protein is also involved in the regulation of telomere replication in humans and whether it is associated with subtelomeric regions.

## Material and Methods

### Cell Culture

For this study, human primary fibroblasts (TP) and an immortalized cell line (TP15.5), derived from these cells, were established as previously described^23^. TP15.5 is a subclone obtained by SV40LT transfection of TP. Cells were grown in DMEM F12 Glutamax (Gibco®, Invitrogen GmbH, Darmstadt, Germany) supplemented with 10% fetal calf serum (Eurobio, Les Ulis, France) and antibiotic antimycotic (Gibco®) at 37 °C in a humidified incubator with an atmosphere containing 5% CO_2_. TP15.5 was cultured for different Population Doublings (PD) from 36 to 703. Telomeric crisis occurred at PD38.

For the telomerase activity studies, cells were stored in dry pellets at −80 °C. For the replication studies, cells were incubated in the presence of 5′-Bromodeoxyuridine (BrdU) (Sigma-Aldrich, St. Louis, MO, USA) at 10 μg/ml during the last 6, 8, 10, 12, 14, 16 and 18 hours of culture. Cells were arrested in mitosis by incubation with colcemid for 2 hours (0.1 μg/ml) before harvesting. The preparation of cytogenetic slides was performed as previously described^44^. Cells were first subjected to hypotonic shock for 15 min in 75 mM potassium chloride at 37 °C, followed by fixation in ethanol/acetic acid and then spread on slides.

### Cytogenetic studies

Different cytogenetic analyses were performed as a function of the type of hybridization. First, for telomere quantification, slides were hybridized with a Protein Nucleic Acid (PNA) telomere probe (CCCTAA)_3_-Cyanine 3 (Cy3) (Panagene, Inc., Daejeon, Korea) as previously described^45^. For karyotyping and/or telomere identification, M-FISH (Multi-Fluorescent *In Situ* Hybridization) and subtelomeric-FISH were performed using multi-FISH probes (MetaSystems Gmbh, Althusseim, Germany) and telomere-specific probes (Cytocell, Cambridge, UK) respectively, according to the manufacturers’ recommendations. For p-arm acrocentric chromosome identification, whole-chromosome painting (MetaSystems) was performed after subtelomeric-FISH. Images of hybridized metaphases were captured with a Zeiss Axioplan II fluorescence microscope and processed using ISIS software (MetaSystems).

### Telomere quantification

Following telomere hybridization, images were captured, using either Metacyte® software (MetaSystems) for global telomere length (around 5000 nuclei, in quadruplate) or Autocapt® software (MetaSystems) for single telomere length (around 30 metaphases). In both cases, quantification was evaluated by grayscale analysis (Q-FISH).

For determining global telomere length, Cy3 intensity was normalized by DAPI (4′6′diamino2-phenylidole) intensity to compensate for the variable number of chromosomes in each PD. The ratio Cy3 intensity/DAPI intensity was then normalized (standard score, normalized value = 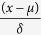 with μ corresponding to the mean and δ to the standard deviation) and adjusted to 100 (a.u.) for TP.

For determining single telomere length, telomeres were identified as described above. Captured images were analyzed using the “Telomere” function in ISIS software. For every metaphase analyzed, each single telomere studied was normalized (standard score). All values defined for each single telomere were regrouped in scatter plots (p value; q value) to distinguish populations corresponding to homologous chromosomes. Then, averages of populations’ values were performed.

### Telomerase activity

To determine telomerase activity, the kit Telo*TAGGG* Telomerase PCR ELISA^PLUS^ (Roche Diagnostics GmbH, Mannheim, Germany) was used. 10 μg of protein of each dry pellet lysate was added to the reaction mixture containing the synthesized telomeres for elongation and PCR steps. The final ELISA step allows the determination of the Relative Telomerase Activity (RTA) of each sample. An internal control (from the kit) and positive control (MDA cells) were used. The experiment was performed four times and the RTA was normalized (standard score) and adjusted to 0 (a.u.) for TP cells.

### ReD-FISH

Replicative Detargeting-FISH (ReD-FISH)^16^ is a FISH technique derived from Chromosome Orientation-FISH (CO-FISH)^25^ using single-stranded PNA probes to produce strand-specific hybridization and was performed as previously described. The technique relies on BrdU incorporation into the newly synthesized DNA strand during a part of S-phase (as opposed to incorporation during the entire S-phase in CO-FISH) before performing the metaphase preparations. After rinsing in 2X SSC (Saline Sodium Citrate), slides were incubated for 15 min in 5 μg/ml Hoechst 33258 (Sigma) that intercalates specifically at BrdU incorporation sites. Slides were then subjected during 45 min to UV irradiation in a 2X SSC bath to induce nicks at Hoechst intercalation sites. The incubation of slides in 10 U/μl ExoIII (Promega, Mannheim, Germany) during 8 min degrades the DNA strand from the nicks. The strands, which incorporate BrdU during the S-phase, will be degraded leaving a part of the parental strand as a single-stranded template for the hybridization procedure. After dehydration in successive baths of 50, 70, and 100% ethanol for 5 min each, slides were incubated for 1h30 with the PNA probe T_2_AG_3_ (FITC, Fluorescein IsoThioCyanate) and then with the probe C_3_TA_2_ (Cyanine 3) for another 1h30. After hybridization and washes, slides were counterstained with DAPI (1 μg/ml).

Thus, telomeres that are replicated after BrdU addition are denoted as detargeted telomeres i.e. one probe per chromatid, whereas those replicated before BrdU incorporation are denoted as non-detargeted telomeres i.e. the two probes per chromatid.

BrdU incorporation during the S-phase allows the identification of the S-sub-phase at the moment the BrdU was added. Thus, for each analyzed cell, we obtain the telomere replication status (replicated/not replicated) for a given S-sub-phase. This is critical because we did not add BrdU as a pulse during a precise moment of S-phase but from a given moment of S-phase (determined with BrDU) until the end of the cycle. We were thus able to detect 5 S-sub-phases and their intervals, thus dividing the replication timing into 4 periods.

### S-phase analysis

BrdU incorporation during the S-phase allowed the identification of the sub-phase, and the determination of the length of the S-phase and the S-sub-phases due to the different incorporation times (the last 6, 8, 10, 12, 14, 16 and 18 hours of culture). Thus, every BrdU incorporation time for ReD-FISH slides of TP and PD64, PD263 and PD523 of TP15.5 was analyzed in reverse DAPI in order to identify the S-sub-phase of each metaphase (around 100 metaphases analyzed per condition) at the moment that BrdU was added in culture. The 5 S-sub-phases, in order, are: early S, early middle S, middle S, late middle S, late S. Thereby, this analysis permits the determination of the length of each of the 5 S-sub-phases, thus the entire S-phase, of each PD studied. Then, for the sake of simplicity, intervals of the 5 S-sub-phases were used, dividing the entire S-phase into 4 periods renamed: early S, early middle S, late middle S and late S. For each PD studied, the mean number of hours was determined for each sub-phase interval.

### ReD-FISH analysis

Telomere replication timing was studied at 8, 10, 12, 14 and 16 hours of BrdU incorporation depending on the PD time of the cells. For each single telomere replication timing analysis, the entire S-phase was divided into 4 periods as described above: early S, early middle S, late middle S and late S (represents the 4 intervals of the 5-S sub-phases). A minimum of 30 metaphases was analyzed for the 4 periods, with the aid of reverse DAPI analysis, for each telomere studied. For the last two periods (late middle S and late S), detargeted telomeres were identified (replication after BrdU incorporation), and for the first two (early S and early middle S), non-detargeted telomeres were identified (replication before BrdU incorporation). The percentage of replication in early S and late S was subtracted from the percentage in early middle S and late middle S respectively for each single telomere to obtain the true percentage of telomere replication during the two periods. Thus the addition of the percentages from all 4 periods was adjusted to 100%. The mean replication timing (mrt) of single telomeres was calculated as follows:


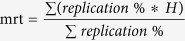


where H corresponds to the mean hour of the sub-phase interval determined in the S-phase analysis (cf. Fig. 2D). Mrt is an indicator of the period in which single telomeres replicate on average.

Every single telomere was analyzed in TP with the aid of M-FISH hybridization as they are normal cells (46XY) with no chromosomal rearrangements contrary to TP15.5. Not all telomeres could be studied in TP15.5 due to the complexity and cost of such study. Thus, telomeres of 9 chromosomes were chosen for study in TP15.5 with the aid of subtelomeric hybridization. These chromosomes were 1; 2; 3; 8; 9; 13; 16; 19; 21. The analysis of these chromosomes permits the study of telomere replication timing of all types of chromosomes i.e. metacentric, submetacentric and acrocentric chromosomes. Each telomere studied for each PD was classified into one of 6 categories from very early S to very late S, according to their mrt. These 6 categories were defined by the length of each sub-phase.

The global flow chart of the analysis is shown in Fig. 3.

### Polymorphism and SV40 hybridization

To distinguish homologous chromosomes, SV40 and polymorphism (F 7501 subtelomeric probe kindly offered by A. Londoño-Vallejo) hybridizations were performed following ReD-FISH. These two hybridization techniques make it possible to distinguish homologous chromosomes 6 and 16. The SV40 plasmid and F7501 cosmid were labeled with DIG (Digoxigenin-11-dUTP) using DIG-NICK Translation Mix (Roche) following the manufacturer’s instructions and then, labelling and *In Situ* Hybridization was performed as described^46^.

Metaphases were retrieved with the aid of the recorded coordinates in MetaSystems software and image capture performed following the second hybridization.

## Additional Information

**How to cite this article**: Piqueret-Stephan, L. *et al.* Replication Timing of Human Telomeres is Conserved during Immortalization and Influenced by Respective Subtelomeres. *Sci. Rep.* **6**, 32510; doi: 10.1038/srep32510 (2016).

## Supplementary Material

Supplementary Information

## References

[b1] Moyzis R. K. *et al.* A highly conserved repetitive DNA sequence, (TTAGGG)n, present at the telomeres of human chromosomes. Proc Natl Acad Sci USA. 85, 6622–6626 (1988). 3413114 10.1073/pnas.85.18.6622 PMC282029

[b2] Blackburn E. H. Switching and signaling at the telomere. Cell. 106, 661–673 (2001). 11572773 10.1016/s0092-8674(01)00492-5

[b3] Wright W. E., Tesmer V. M., Huffman K. E., Levene S. D. & Shay J. W. Normal human chromosomes have long G-rich telomeric overhangs at one end. Genes Dev. 11, 2801–2809 (1997). 9353250 10.1101/gad.11.21.2801 PMC316649

[b4] Griffith J. D. *et al.* Mammalian telomeres end in a large duplex loop. Cell. 97, 503–514 (1999). 10338214 10.1016/s0092-8674(00)80760-6

[b5] Wellinger R. J.& Sen D. The DNA structures at the ends of eukaryotic chromosomes. Eur J Cancer. 33, 735–749 (1997). 9282112 10.1016/S0959-8049(97)00067-1

[b6] Mefford H. C. & Trask B. J. The complex structure and dynamic evolution of human subtelomeres. Nat Rev Genet. 2, 91–102 (2002). 11836503 10.1038/nrg727

[b7] Riethman H., Ambrosini A. & Paul S. 1. Human subtelomere structure and variation. Chromosome Res. 5, 505–515 (2005). 16132815 10.1007/s10577-005-0998-1

[b8] Levy M. Z., Allsopp R. C., Futcher A. B., Greider C. W.& Harley C. B. Telomere end-replication problem and cell aging. J Mol Biol. 225, 951–960 (1992). 1613801 10.1016/0022-2836(92)90096-3

[b9] Hayflick L. The limited *in vitro* lifetime of human diploid cell strains. Exp Cell Res. 37, 614–636 (1965). 14315085 10.1016/0014-4827(65)90211-9

[b10] Olovnikov A. M. A theory of marginotomy. The incomplete copying of template margin in enzymic synthesis of polynucleotides and biological significance of the phenomenon. J Theor Biol. 41, 181–190 (1973). 4754905 10.1016/0022-5193(73)90198-7

[b11] Greider C. W. & Blackburn E. H. Identification of a specific telomere terminal transferase activity in Tetrahymena extracts. Cell. 43, 405–413 (1985). 3907856 10.1016/0092-8674(85)90170-9

[b12] Stewart S. A. & Weinberg R. A. Telomeres: cancer to human aging. Annu Rev Cell Dev Biol. 22, 531–557 (2006). 16824017 10.1146/annurev.cellbio.22.010305.104518

[b13] Bryan T. M., Englezou A., Dalla-Pozza L., Dunham M. A. & Reddel R. R. Evidence for an alternative mechanism for maintaining telomere length in human tumors and tumor-derived cell lines. Nat Med. 3, 1271–1274 (1997). 9359704 10.1038/nm1197-1271

[b14] Murnane J. P., Sabatier L., Marder B. A. & Morgan W. F. Telomere dynamics in an immortal human cell line. EMBO J. 20, 4953–4962 (1994). 7957062 10.1002/j.1460-2075.1994.tb06822.x PMC395436

[b15] Wright W. E., Tesmer V. M., Liao M. L. & Shay J. W. Normal human telomeres are not late replicating. Exp Cell Res. 251, 492–499 (1999). 10471333 10.1006/excr.1999.4602

[b16] Zou Y., Gryaznov S. M., Shay J. W., Wright W. E. & Cornforth M. N. Asynchronous replication timing of telomeres at opposite arms of mammalian chromosomes. Proc Natl Acad Sci USA. 101, 12928–12933, Epub 2004 Aug 20 (2004). 15322275 10.1073/pnas.0404106101 PMC516496

[b17] Arnoult N. *et al.* Replication timing of human telomeres is chromosome arm-specific, influenced by subtelomeric structures and connected to nuclear localization. PLoS Genet. 6(4), e1000920, doi: 10.1371/journal.pgen.1000920 (2010). 20421929 PMC2858680

[b18] McCarroll R. M. & Fangman W. L. Time of replication of yeast centromeres and telomeres. Cell. 54, 505–513 (1988). 3042152 10.1016/0092-8674(88)90072-4

[b19] Bianchi A. & Shore D. Early replication of short telomeres in budding yeast. Cell. 128, 1051–1062 (2007). 17382879 10.1016/j.cell.2007.01.041

[b20] Gilson E. & Géli V. How telomeres are replicated. Nat Rev Mol Cell Biol. 8, 825–838 (2007). 17885666 10.1038/nrm2259

[b21] Cosgrove A. J., Nieduszynski C. A. & Donaldson A. D. Ku complex controls the replication time of DNA in telomere regions. Genes Dev. 16, 2485–2490 (2002). 12368259 10.1101/gad.231602 PMC187453

[b22] Novo C. *et al.* The heterochromatic chromosome caps in great apes impact telomere metabolism. Nucleic Acids Res. 41, 4792–4801 (2013). 23519615 10.1093/nar/gkt169 PMC3643582

[b23] Ducray C., Pommier J. P., Martins L., Boussin F. D. & Sabatier L. Telomere dynamics, end-to-end fusions and telomerase activation during the human fibroblast immortalization process. Oncogene. 18, 4211–4223 (1999). 10435634 10.1038/sj.onc.1202797

[b24] Hanahan D. & Weinberg R. A. Hallmarks of cancer: the next generation. Cell. 144, 646–674 (2011). 21376230 10.1016/j.cell.2011.02.013

[b25] Bailey S. M., Goodwin E. H. & Cornforth M. N. Strand-specific fluorescence *in situ* hybridization: the CO-FISH family. Cytogenet Genome Res. 107, 14–17 (2004). 15305050 10.1159/000079565

[b26] Bianchi A. & Shore D. How telomerase reaches its end: mechanism of telomerase regulation by the telomeric complex. Mol Cell. 31, 153–165 (2008). 18657499 10.1016/j.molcel.2008.06.013

[b27] Hemann M. T., Strong M. A., Hao L. Y. & Greider C. W. The shortest telomere, not average telomere length, is critical for cell viability and chromosome stability. Cell. 107, 67–77 (2001). 11595186 10.1016/s0092-8674(01)00504-9

[b28] Marcand S., Gilson E. & Shore D. A protein-counting mechanism for telomere length regulation in yeast. Science. 275, 986–990 (1997). 9020083 10.1126/science.275.5302.986

[b29] Ouellette M. M. *et al.* Subsenescent telomere lengths in fibroblasts immortalized by limiting amounts of telomerase. J Biol Chem. 275, 10072–10076 (2000). 10744686 10.1074/jbc.275.14.10072

[b30] Samper E., Flores J. M. & Blasco M. A. Restoration of telomerase activity rescues chromosomal instability and premature aging in Terc-/- mice with short telomeres. EMBO Rep. 2, 800–807 (2001). 11520856 10.1093/embo-reports/kve174 PMC1084029

[b31] Steinert S., Shay J. W. & Wright W. E. Transient expression of human telomerase extends the life span of normal human fibroblasts. Biochem Biophys Res Commun. 273, 1095–1098 (2000). 10891377 10.1006/bbrc.2000.3080

[b32] Zhu L. *et al.* Telomere length regulation in mice is linked to a novel chromosome locus. Proc Natl Acad Sci USA. 95, 8648–8653 (1998). 9671732 10.1073/pnas.95.15.8648 PMC21130

[b33] Londoño-Vallejo J. A., DerSarkissian H., Cazes L. & Thomas G. Differences in telomere length between homologous chromosomes in humans. Nucleic Acids Res. 29, 3164–3171 (2001). 11470873 10.1093/nar/29.15.3164 PMC55832

[b34] Hastie N. D. *et al.* Telomere reduction in human colorectal carcinoma and with ageing. Nature. 346, 866–868 (1990). 2392154 10.1038/346866a0

[b35] Slagboom P. E., Droog S. & Boomsma D. I. Genetic determination of telomere size in humans: a twin study of three age groups. Am J Hum Genet. 55, 876–882 (1994). 7977349 PMC1918314

[b36] Heride C. *et al.* Distance between homologous chromosomes results from chromosome positioning constraints. J Cell Sci. 123, 4063–4075 (2010). 21084563 10.1242/jcs.066498

[b37] Drosopoulos W. C., Kosiyatrakul S. T., Yan Z., Calderano S. G. & Schildkraut C. L. Human telomeres replicate using chromosome-specific, rather than universal, replication programs J Cell Biol. 197, 253–266 (2012). 22508510 10.1083/jcb.201112083 PMC3328383

[b38] Yamazaki S. *et al.* Rif1 regulates the replication timing domains on the human genome. EMBO J. 31, 3667–3677 (2012). 22850674 10.1038/emboj.2012.180 PMC3442267

[b39] Yamazaki S., Hayano M. & Masai H. Replication timing regulation of eukaryotic replicons: Rif1 as a global regulator of replication timing. Trends Genet. 29, 449–460 (2013). 23809990 10.1016/j.tig.2013.05.001

[b40] Mattarocci S. *et al.* Rif1 controls DNA replication timing in yeast through the PP1 phosphatase Glc7. Cell Rep. 7, 62–69, doi: 10.1016/j.celrep.2014.03.010 (2014). 24685139

[b41] Davé A., Cooley C., Garg M. & Bianchi A. Protein phosphatase 1 recruitment by Rif1 regulates DNA replication origin firing by counteracting DDK activity. Cell Rep. 7, 53–61, doi: 10.1016/j.celrep.2014.02.019 (2014). 24656819 PMC3989773

[b42] Hiraga S. *et al.* Rif1 controls DNA replication by directing Protein Phosphatase 1 to reverse Cdc7-mediated phosphorylation of the MCM complex. Genes Dev. 28, 372–383, doi: 10.1101/gad.231258.113 (2014). 24532715 PMC3937515

[b43] Foti R. *et al.* Nuclear Architecture Organized by Rif1 Underpins the Replication-Timing Program. Mol Cell. 21, 260–273, doi: 10.1016/j.molcel.2015.12.001,Epub 2015 Dec 24 (2016). 26725008 PMC4724237

[b44] IAEA. “Cytogenetic dosimetry: Applications in preparedness for and response to radiation emergencies”, In Emergency Preparedness and Response Series. (Vienna, 2011).

[b45] Lansdorp P. M. *et al.* Heterogeneity in telomere length of human chromosomes. Hum Mol Genet. 5, 685–691 (1996). 8733138 10.1093/hmg/5.5.685

[b46] Viegas-Pequignot E., Dutrillaux B., Magdelenat H. & Coppey-Moisan M. Mapping of single-copy DNA sequences on human chromosomes by *in situ* hybridization with biotinylated probes: enhancement of detection sensitivity by intensified-fluorescence digital-imaging microscopy. Proc Natl Acad Sci USA. 86, 582–586 (1989). 2643118 10.1073/pnas.86.2.582 PMC286516

