# Supplementary material for: Replication Timing of Human Telomeres is Conserved during Immortalization and Influenced by Respective Subtelomeres
Source: Sci Rep. 2016 Sep 2;6:32510. doi: 10.1038/srep32510 (PMC5009427; doi:10.1038/srep32510)
Supplement: Supplementary Information [file srep32510-s1.pdf]

Replication Timing of Human Telomeres is Conserved during Immortalization and Influenced by Respective Subtelomeres

Running title: Subtelomeres Govern Telomere Replication Timing

Laure Piqueret-Stephan¶, Michelle Ricoul¶, William M. Hempel, Laure Sabatier*

¶ These two authors contribute equally to this work.
* Corresponding author: Dr Laure Sabatier, e-mail: laure.sabatier@cea.fr , phone: +33146548344

PROCyTOX Commissariat à l'Energie Atomique et aux Energies Alternatives (CEA), Fontenay-aux-Roses and Université Paris-Saclay, France.

Supplemental figures S1, S2 and S3 (in order of apparition)

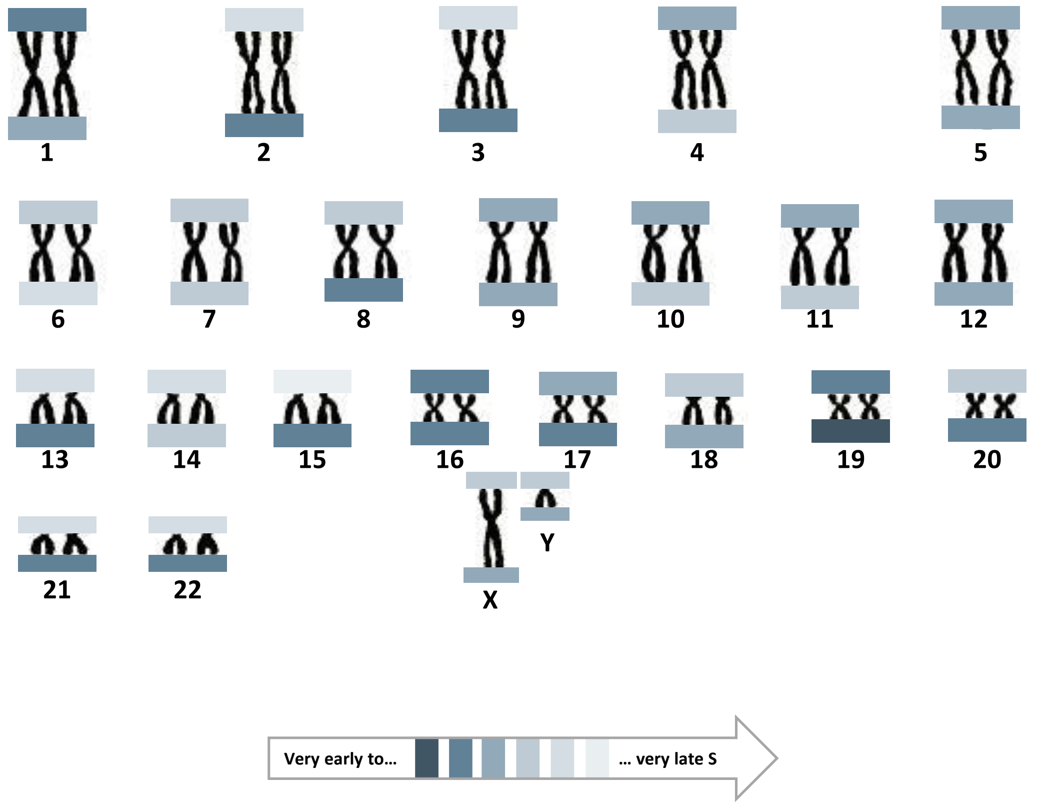
1
2
3
4
5
6
7
8
9
10
11
12
13
14
15
16
17
18
19
20
21
22
X
Y
Very early to...
... very late S

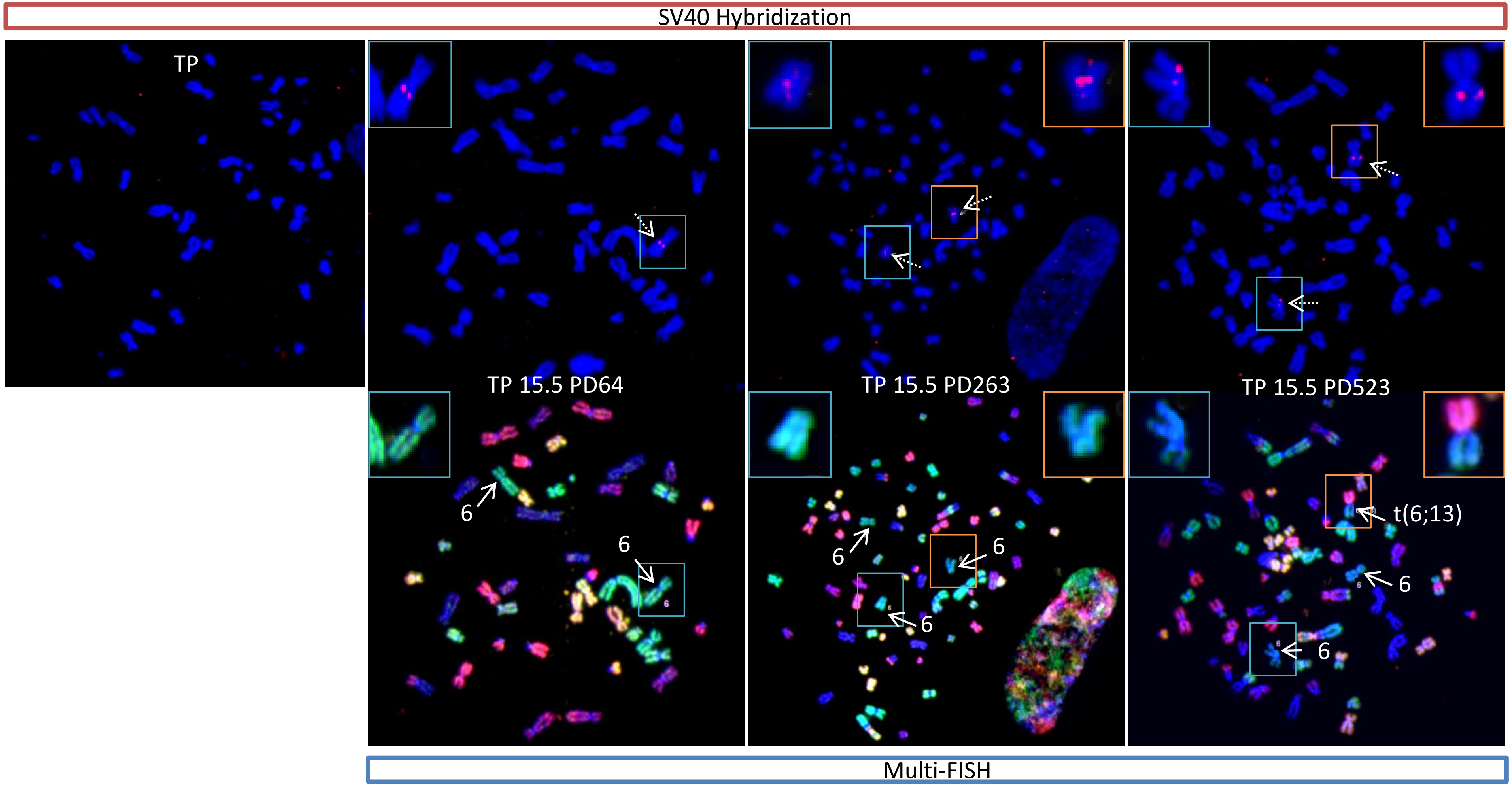
SV40 Hybridization
TP
TP 15.5 PD64
TP 15.5 PD263
TP 15.5 PD523
6
6
6
6
6
6
6
t(6;13)
6
6
Multi-FISH

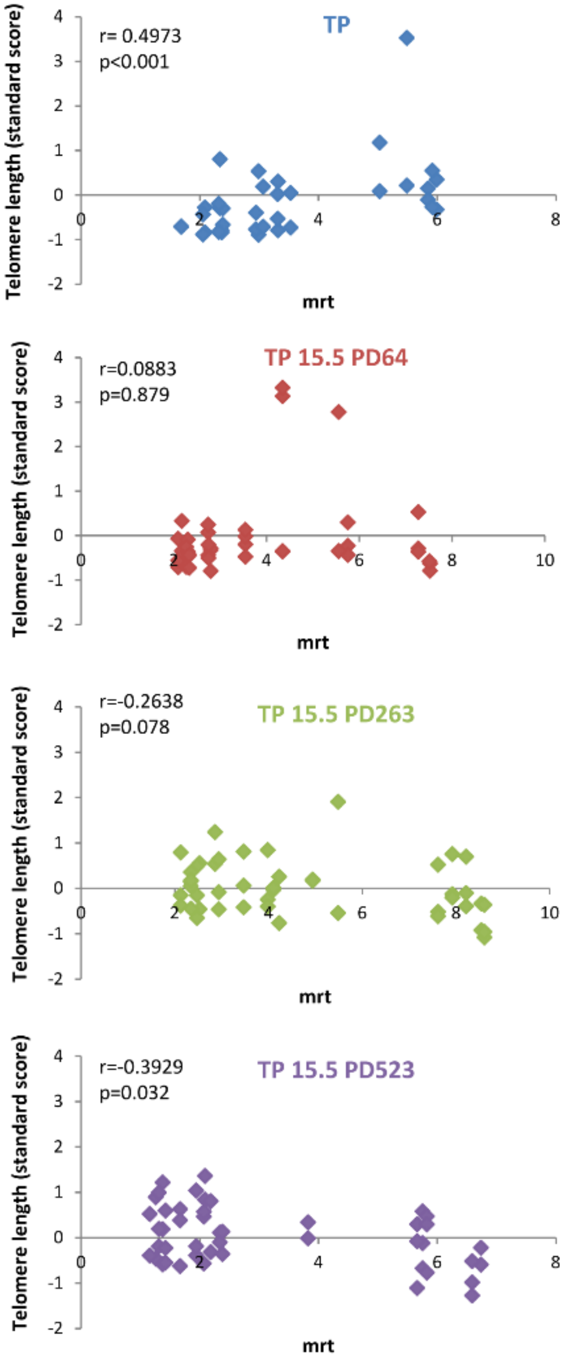
TP
r= 0.4973
p<0.001
TP 15.5 PD64
r=0.0883
p=0.879
TP 15.5 PD263
r=-0.2638
p=0.078
TP 15.5 PD523
r=-0.3929
p=0.032
Telomere length (standard score)
mrt

Supplemental figure legends

- **Figure S1. Summary of telomere replication timing in the control cell TP.** Telomeres are classified according to their mrt as described above. Only two telomeres are replicated on average at both ends of S-phase: 19q in very early S and 15p in very late S. Replication of the 46 other telomeres is distributed throughout the other four categories.

- **Figure S2. After transfection, SV40 was integrated into chromosome 6.** SV40 plasmid hybridization was carried out in TP as a negative control cell and in the three PD studied in TP15.5 (i.e. PD64, 263 and 523) (top panels). The plasmid was revealed using antibodies labeled with TexasRed. Chromosomes which have integrated the SV40 plasmid are indicated by the dotted arrows. Multi-FISH was then carried out in SV40-positive cells to identify the chromosomes into which the plasmid integrated (bottom panels). Chromosome 6 was the only chromosome integrated by SV40 after transfection. All copies of chromosome 6 of each metaphase are indicated by full arrows. Just after crisis (PD64) SV40 is integrated into one of the two copies of chromosome 6. Later, after replication (PD263 and 523), when polyploidy is established, two copies of chromosome 6 out of the three chromosomes is integrated by SV40, indicating that one out of the two SV40-integrated chromosomes is the duplicate of the other. One of these chromosomes is rearranged in a clonal translocation t(6;13).

- **Figure S3. Single telomere replication timing does not appear to correlate with their length.** No correlation between the mrt and telomere length in the studied PD (TP in blue, TP15.5 PD64 in red, TP15.5 PD263 in green and TP15.5 PD523 in purple). The correlation coefficients are too weak to suggest any correlation even if the p-value of TP and TP15.5 PD523 is significant. Indeed, telomere length varies during immortalization whereas telomere replication does not (Spearman’s rank correlation coefficient was used).
